# Colostrum Quality in Different Goat Breeds Reared in Northern Italy

**DOI:** 10.3390/ani13193146

**Published:** 2023-10-09

**Authors:** Stella Agradi, Marta González-Cabrera, Anastasio Argüello, Lorenzo Enrique Hernández-Castellano, Noemí Castro, Laura Menchetti, Gabriele Brecchia, Daniele Vigo, Edoardo Tuccia, Giulio Curone

**Affiliations:** 1 Department of Veterinary Medicine and Animal Sciences, University of Milan, Via dell’Università 6, 26900 Lodi, Italy; stella.agradi@unimi.it (S.A.); daniele.vigo@unimi.it (D.V.); giulio.curone@unimi.it (G.C.); 2 IUSA-ONEHEALTH 4. Animal Production and Biotechnology, Institute of Animal Health and Food Safety, Universidad de Las Palmas de Gran Canaria, Campus Montaña Cardones, s/n, 35413 Arucas, Spain; marta.gonzalezcabrera@ulpgc.es (M.G.-C.); tacho@ulpgc.es (A.A.); lorenzo.hernandez@ulpgc.es (L.E.H.-C.); noemi.castro@ulpgc.es (N.C.); 3 School of Biosciences and Veterinary Medicine, University of Camerino, Via Circonvallazione 93/95, 62024 Matelica, Italy; 4 ET Dairy Veterinary Services, Via Magenta 14, 26900 Lodi, Italy; edoardo.tuccia@gmail.com

**Keywords:** biodiversity, small ruminants, immunoglobulins, lactoferrin, local breed

## Abstract

**Simple Summary:**

The progressive abandonment of local breeds dramatically threatens livestock biodiversity. The knowledge of these breeds should be broadened to understand their adaptive strategies, optimize their performance, promote their conservation, and thus contribute to ecological restoration. Investigating colostrum quality could be meaningful for understanding the nutritive concentration capacities and immunological status of females, and, as a consequence, for the future health status and growth of newborns. We hypothesize that the composition of goat colostrum is influenced by the breed and its typical farming system making colostrum from local breeds different from cosmopolitan ones. This study aimed to characterize colostrum quality, including basic chemical composition (i.e., fat, protein, lactose, and total solids) and immune variables (i.e., IgG, IgM, and lactoferrin) in three different local goat breeds from Northern Italy (i.e., Frisa Valtellinese, Orobica, and Lariana) and a cosmopolitan one (i.e., Camosciata delle Alpi) reared under traditional semi-extensive and intensive systems, respectively. Results showed variability in the colostrum quality among breeds, which could be linked to the different farming systems, processes of artificial and natural selection, and meat or dairy aptitude of the animals. However, local goats had a higher quality of colostrum that could confer greater hardiness and rusticity to their kids.

**Abstract:**

This study aimed to characterize the colostrum quality in three different local goat breeds of Northern Italy (i.e., Frisa Valtellinese, Orobica, and Lariana) and a cosmopolitan one (i.e., Camosciata delle Alpi) (*n* = 30 per breed), reared under traditional semi-extensive and intensive systems, respectively. Lariana showed the highest percentage of fat (10.18 ± 3.14%) and total solids (30.73 ± 4.89%) but the lowest percentage of lactose (1.87 ± 0.82%; *p* < 0.05); Orobica had the lowest percentage of fat (7.13 ± 2.48%), total solids (24.11 ± 5.48%), and protein (10.77 ± 4.53%) but the highest percentage of lactose (3.16 ± 0.73%; *p* < 0.05). This suggests that breeds which have a more pronounced meat aptitude (i.e., Frisa and Lariana) have a higher concentration of components than breeds with more dairy aptitude (i.e., Orobica and Camosciata). Uni- and multivariate analyses showed that IgG is the parameter that best differentiates local breeds from cosmopolitan ones (*p* < 0.01). Colostrum from Frisa goats showed the highest IgG concentration (100.90 ± 8.11 mg/mL), while the lowest concentration was in the Camosciata breed (74.75 ± 20.16 mg/mL). Finally, the highest lactoferrin concentration was in Frisa (1781.3 ± 892.6 µg/mL) and the lowest in Camosciata and Lariana (763.1 ± 357.9 and 1148.0 ± 858.6 µg/mL, respectively; *p* < 0.05). Differences between Camosciata and local breeds could be due to the different farming systems, in addition to the genetic characteristics. The higher quality of colostrum produced by some local goats could be an adaptive characteristic that helps the growth and survival of the kids.

## 1. Introduction

Autochthonous livestock breeds are important as genetic resources of variability [1,2]. Italy, due to its peculiar and diversified pedogeographical characteristics, has given rise over the centuries to the selection of numerous breeds [3,4,5]: nearly 40 goat breeds have been recognized in the constitution of a Genealogical Book [6]. Despite their valuable multifunctional role, including the maintenance of biodiversity, most of the local caprine breeds are facing extinction. This is due to the progressive abandonment of these rustic but low-productive goats in favor of cosmopolitan breeds which are more productive and suitable for intensive farming [7,8]. For this reason, it is essential to study the physiology and ethology as well as the productive characteristics of the autochthonous breeds, to understand their adaptive strategies, and to encourage their use and conservation.

One of the most essential physiological mechanisms for the survival and development of goat kids is based on colostrum intake during the first hours of life. Colostrum is the first secretion of the mammary gland that the mother secretes after parturition. It provides vital nutrients to newborns which enhance their natural defenses and regulates immune response, gut microbiota, and growth and repair of tissues [9,10,11]. In fact, the molecules and immune cells contained in colostrum are very important in allowing the transfer of passive immunity and the creation of an initial protective barrier in the newborn’s intestine and other mucus membranes [11]. These, however, are only some of the main functions performed by colostrum. In goats, colostrum composition has been investigated since 1840 in different breeds worldwide [12]. Studies have characterized the colostrum of German, Swiss [13,14], Spanish [15,16,17,18], and cosmopolitan breeds [19,20,21]. On the other hand, Italian breeds have been neglected. Just a few studies have analyzed the colostrum of the Garganica goat, an indigenous breed of Southern Italy, and only as regards its oligosaccharides content [22,23]. In general, goat colostrum is characterized by a dry matter above 20% with a high and variable percentage of fat (on average 8%) mainly constituted by short-chain fatty acids which are easily digested and absorbed by the newborn. In addition, it is also characterized by a high percentage of protein (usually above 10%) with a high biological titer and a relatively low percentage of lactose (around 2%). Not only does colostrum provide energy to the newborn mammal, but it also contains very important bioactive components that play a key role in passive immune transfer. The particular anatomical conformation of the goat placenta does not allow a sufficient transfer of immune components, mainly immunoglobulins, from the does to the fetus. Therefore, kids are born agammaglobulinemic and unable to cope with possible infections [24]. Thus, ruminants’ colostrum is particularly rich in immunoglobulins (Ig), which make up one-third of the total colostrum proteins. In goat colostrum, IgG is the main immunoglobulin accounting for 90% of the total immunoglobulins, followed by IgM (6.0%) and finally IgA (3.7%) [13]. In addition to these protein molecules, other factors such as vitamins, hormones, growth factors, cytokines, enzymes and bioactive peptides, and immunocompetent cells act as adjuvants to the kid’s immune system [24,25,26]. These include lactoferrin, an iron-binding glycoprotein, which makes metal ions unavailable to pathogens, inhibiting their growth. It is one of the most emphasized immune-stimulating factors of the bovine colostrum [27,28], but its content in the goat colostrum is still little known [29,30]. The quality of colostrum may depend on various factors. Parity and litter size could influence it, but findings are still controversial [13,14,15,16,17,18,19,21,24,31], while regarding the farming system, studies have been carried out only in cattle [32]. Moreover, variations in the colostrum composition occur not only according to the species, but also to the breed, and, to our knowledge, there is no information concerning the local breeds of Northern Italy.

Specifically, within the zootechnical context of Northern Italy, the goats most commonly reared for milk and cheese production are two cosmopolitan breeds, the Camosciata Alpina or Alpine and the Saanen goats. These transboundary breeds are usually farmed with an intensive or semi-intensive system, which enables the genetic base artificially selected for better production performance to be fully exploited. In these contexts, the environment where the animals are bred, and the administered diet are controlled and standardized. The local breeds, although in the minority, are still widespread in the pre-Alpine and Alpine areas in small flocks [33]. Frisa Valtellinese, Orobica, and Lariana are among the most important autochthonous breeds in Northern Italy, where they are used for the production of typical products, especially fresh or seasoned cheeses, and kid meat. These breeds are farmed according to the traditional system, based on a close environment-(domestic) animal-human link. In fact, during the winter months, the animals are housed indoors and fed with hay, while usually in spring and autumn, intermittent grazing is carried out in the areas surrounding the farms. Finally, in the summer months, the traditional vertical transhumance is performed, whereby the flocks are moved to high altitudes to graze on alpine pastures [34]. Few studies have involved the caprine heritage of these areas, especially as regards local breeds. Defining the quality of colostrum could be significant for understanding the nutritive concentration capacities and immunological status of the females, and, as a consequence, the future health status and growth of the newborns [20]. Moreover, colostrum can be considered a nutraceutical that can potentially provide beneficial effects on human [35,36,37] and animal health [38,39,40]. Thus, studies on the composition of the goat colostrum could be also useful to produce supplements that could be used in human and animal medicine.

We hypothesize that the composition of colostrum is influenced by breed and that local breeds may have similar characteristics, due to the similar farming system and evolutionary processes, that distinguish them from the cosmopolitan one. The composition of colostrum could be thus a trait related to the goat’s adaptability and contribute to the greater rusticity of the autochthonous breeds. This study aimed to characterize the colostrum quality in three different local goat breeds of Northern Italy (i.e., Frisa Valtellinese, Orobica, and Lariana), and a cosmopolitan one (i.e., Camosciata delle Alpi or Alpine), reared under traditional semi-extensive and intensive systems, respectively, both from a basic composition (fat, protein, lactose, and total solids percentages) and immunological (IgG, IgM, and lactoferrin concentrations) point of view.

## 2. Materials and Methods

### 2.1. Animals Enrolled

In this investigation, 120 adult female goats were enrolled (i.e., Frisa, *n* = 30; Orobica, *n* = 30; Lariana, *n* = 30; and Camosciata delle Alpi, *n* = 30).

**Figure 1:**
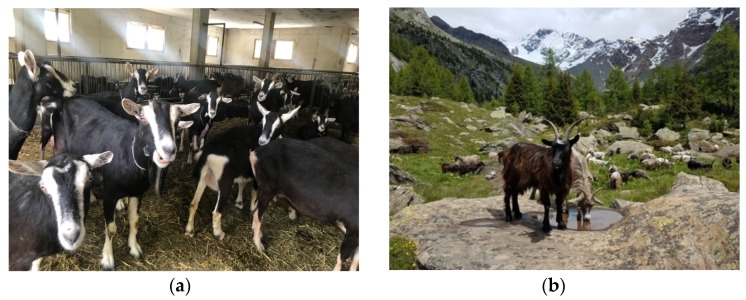

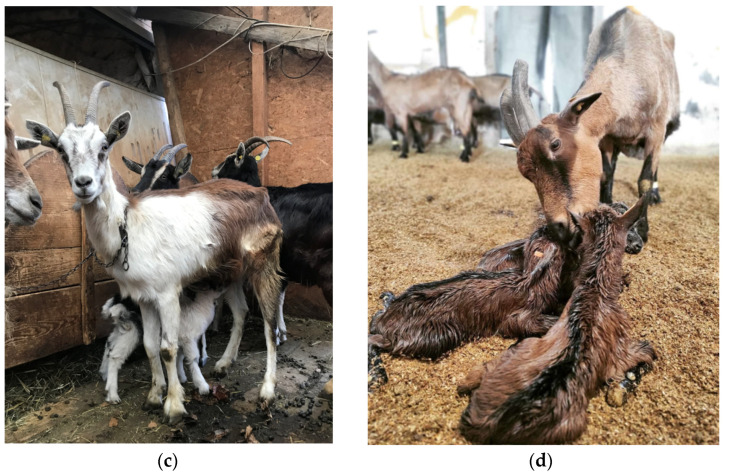
Frisa Valtellinese (**a**), Orobica (**b**), Lariana (**c**), and Camosciata delle Alpi goats (**d**) in their respective breeding environment in different times of the year.

The Frisa or Frontalasca is a dual-purpose goat (Figure 1a) native to the Rezzalo valley, in North Lombardy. It is mainly reared in Valtellina, Malenco Valley, Masino Valley, Valchiavenna, Valcamonica, Bergamo Valleys, and in the Lario area [41]. Its most relevant morphologic characteristics are its black coat (short or medium length hair) with white stripes on both sides of the head up until the ears; its white hair on the ventral part of the abdomen, on the limb distal extremities, and under the tail; and its ibex type horns (even if polled subjects may be seen) [42]. It is a large-sized goat, well-proportioned, and with a strong constitution that also allows it to exploit the most difficult pastures [41]. The Anagraphic Registry was instituted in 1997. In 2016, the registered population was 785 heads in 51 farms [6]. This goat breed is mainly reared to produce typical cheeses and meat, especially the traditional Valchiavenna goat Violino, which is made by seasoning the legs and shoulders of adult goats.

The Orobica or Valgerola goat (Figure 1b) is an autochthonous breed reared in the Lombardy region in Northern Italy. Its origin is unknown, even if some hypotheses about a Southern Italian provenance (also supported by the oral tradition of old local breeders) have been formulated [42,43]. Nowadays, this breed can be found in the Orobic Prealps in small farms mainly in the Sondrio, Como, Lecco, and Bergamo provinces and, in particular, in Val Gerola, Valsassina, Upper East Lario, and Val Brembana [41]. Morphologically, it is a medium size goat, well-proportioned and adapted to the mountain pasture [44]. The animal is characterized by the presence in both sexes of long horns with a slight helical twist and by four long hair coats which differ in color and pigment distribution (Farinel, Marin, Nigru and Camosch) [44], characteristics that allow their differentiation from other alpine goat breeds and that testify to the probable different origin of Orobica [42]. The Genealogical Book was activated in 1992, and at the end of 2016, the registered population was 1294 heads in 93 farms [6]. It is a dual-purpose breed, with particularly important cheese production. Indeed, the milk is mainly utilized to produce Valtellina’s Bitto cheese, Storico Ribelle cheese (both with at most 10% goat milk), and Orobica goat cheeses (100% Orobica goat milk) [45].

The Lariana or di Livo goat (Figure 1c) is another Lombard autochthonous breed mainly reared in the Livo Valley and Western Lario whose origin is unknown [41]. It is classified as a traditional or primary population because of its highly phenotypic polymorphism [46]. It is a medium–large size goat with a strong constitution [41]. Being that this breed has not undergone a selection for the phenotypic characteristics, morphologically it shows a wide variety of coat colors, hair lengths, and horn types [46]. The Anagraphic Registry was activated in 2001, and at the end of 2016, the registered population was 615 heads in 50 farms [6]. It is mainly bred to produce cheeses and meat.

The farming system is the traditional one for all three of these local breeds [34]. They are very rustic animals, adapted to the Alpine environment where they have been selected for centuries.

The Camosciata delle Alpi or Alpine goat (Figure 1d) is a transboundary dairy breed native to Switzerland, from the cantons of Bern, Freiburg, Glarus, and Graubünden in a mountainous region. It is bred in different countries of the world, mainly in Europe but also in other continents such as North America. This breed is a medium–large size goat, with a typical short hair fawn coat, black dorsal stripe and extremities of limbs, and a characteristic facial mask. They have ibex-type horns both in males and females. In Italy, the Genealogical Book was activated in 1973. To date, the breed is subjected to accurate genetic selection to improve quantitative and qualitative milk and cheese production [6]. Artificial selection in the past decades has led, as in the bovine sector, to the production of less rustic animals, which are less suitable for the semi-extensive farming system and to the exploitation of extreme environments such as the alpine one.

The goats enrolled in the study belonged to 4 different farms according to the breed. The Frisa farm was in Val Bregaglia in the Sondrio province, the Orobica farm was at the beginning of Valtellina in the Sondrio province, and the Lariana farm was in Valle Albano in the Como province. The traditional farming system implied indoor housing from mid-November till mid-May with administration of polyphyte hay ad libitum. During the spring and autumn months, at the beginning and at the end of the indoor housing period, the goats were left free to graze in the vicinity of their respective farms for a limited number of hours/day in order to get used to the fresh forage before vertical transhumance in spring and to get used to a hay-based diet in winter. Vertical transhumance was performed in mid-May to the respective alpine pasture for all farms. The respective alpine pasture areas were located in Val Bregaglia at around 1400–2000 m.s.l., in Val Masino at around 1600–2700 m.s.l., and in Valle Albano at around 1100–1600 m.s.l. All 3 farms kept between 50 and 100 lactating goats only for the production of cheese under a semi-extensive system, following the alpine traditional farming system mentioned above. The Camosciata delle Alpi farm was in the Lodi province in South Lombardy and bred around 100 lactating goats under an intensive system for both the production of milk and cheese. They received a unified diet during the dry period (feed consisted of polyphyte hay, maize, soya, hulled sunflower seed meal, extruded linseed, cane molasses, carob, mineral vitamin corrector), specially designed to reach the energetic requirements according to the physiological stage of the animal. For all breeds, reproduction occurred with natural mating, and the birth season took place from the last week of December till the last week of February 2021. All the goats were milked twice a day after birth, with a milking machine for Camosciata delle Alpi and Frisa during the indoor housing period, and by hand for Frisa during the alpine pasture period and for Orobica and Lariana for the whole lactation. Lactation lasted around 8 and 7 months for Camosciata delle Alpi and Frisa, respectively, while it lasted just 6 months for Orobica and Lariana.

All the goats enrolled in the study were submitted to a clinical evaluation before starting the investigation, and only healthy animals were selected. Since major differences related to the parity order may arise among primiparous and multiparous [17,47], nulliparous goats were not included in this study to minimize the possible bias. The parity order ranged from 1st to 10th (not considering the birth of the kidding season we investigated), while the mean (and standard deviation) age of enrolled goats was 5.6 ± 2.7, 4.9 ± 2.1, 5.4 ± 3.1, and 3.0 ± 1.1 years for Frisa, Orobica, Lariana, and Camosciata delle Alpi goats, respectively. Litter size ranged from 1 to 3 kids (Table S1).

### 2.2. Colostrum Collection

Within 6 h of parturition, before kids began suckling, 50 mL of colostrum were collected by hand milking from both mammary glands in 50 mL Falcon^®^ tubes (Corning, Corning, NY, USA) from 30 adult goats per breed. The samples were immediately frozen at −20 °C and stored until analysis. The collection of colostrum samples from live animals was performed in respect of animal welfare according to current legislation. The study was conducted with the approval of the Institutional Animal Care and Use Committee of Università degli Studi di Milano (Permission OPBA_04_2021). Samples that were insufficient in quantity for analysis, and samples derived from goats that developed any pathology during the peri-partum period, were discarded. For these reasons, a total of 102 samples were analyzed (i.e., 29, 18, 27, and 28 samples for Frisa, Orobica, Lariana, and Camosciata delle Alpi, respectively).

### 2.3. Colostrum Analysis

The basic composition (i.e., fat, protein, lactose, and total solids percentages) was determined by MilkoScan Mars™ (FOSS Analytical A/S, Hillerød, Denmark). For this analysis, colostrum samples were diluted 1:1 (*v*/*v*) using saline solution. Immunoglobulins G, IgM, and lactoferrin concentrations were measured using ELISA commercial kits (Bethyl Laboratories, Montgomery, TX, USA and MyBioSource Inc., San Diego, CA, USA, respectively). All determinations were carried out in duplicate. The intra- and inter-assay coefficients of variation (CV) were <6% and <5% for IgG, <3% and <5% for IgM, and <10% and <10% for the lactoferrin kit.

### 2.4. Statistical Analysis

Diagnostic graphs, Kolmogorov-Smirnov, and Levene’s tests were used to verify assumptions. Two outliers were eliminated (i.e., 1 for IgG and 1 for fat), while lactoferrin was ln-transformed to improve data distribution. Descriptive statistics were used to present variables as means and standard error of the mean [8]. Then, the effect of breed on colostrum composition was first investigated using ANCOVA via Generalized Linear Models (GLMs) Univariate procedures including the parity order and litter size as covariates. The assumption of independence of the covariate and breed effect was verified by checking whether breeds differ in parity order and litter size [48] using, respectively, Kruskal-Wallis and Fisher’s exact tests. These tests showed the lack of independence between breed and litter size since the Camosciata had a greater proportion of twins than local breeds (*p* < 0.001; Table S1). Therefore, the litter size could not be included as a covariate in the ANCOVAs. On the other hand, GLMs including parity order as covariate, breed as fixed factor, and colostrum components as a dependent variable showed that parity order was never significant (*p* > 0.1 for all the variables). Finally, we decided to exclude them and use the classic procedures of the 1-way ANOVA where only the breed effect was evaluated. Welch’s F was used for IgG, IgM, ln-lactoferrin, protein, and total solids as the homogeneity of variance assumption was not met. To highlight the differences between the cosmopolitan and local breeds and farming systems, the contrast “Camosciata vs. [(Frisa + Orobica + Lariana)/3]” was planned. Moreover, pairwise comparisons were performed using Sidak correction.

Subsequently, breeds were categorized into a dichotomous variable according to the breed type (local and cosmopolitan) and farming system (traditional semi-extensive and intensive systems). The two groups included Camosciata (ID = 1) and Frisa, Orobica, and Lariana (ID = 2). Then, all colostrum components were included in a discriminant analysis (DA). DA is a multivariate technique intended to identify the colostrum components that distinguish the two groups (i.e., local breeds from the cosmopolitan one) and quantify their relative importance [49,50]. The coefficients of the discriminant function (Df) were adjusted for group size. The relative importance of each component in classifying the goat’s group (i.e., cosmopolitan vs. local breeds) was evaluated by using Wilks’ lambda (λ, the smaller the Wilks’ λ score, the more important the variable to the Df) and by the structure coefficient (which assesses the importance of each independent variable’s unique contribution to the discriminant function) [49].

Statistical analyses were performed with SPSS 25.0 (SPSS Inc., Chicago, IL, USA) and statistical significance occurred when *p* < 0.05.

## 3. Results

The breed effect on colostrum composition was significant for all components analyzed except IgM (*p* ≤ 0.001; Table 1). Results of pairwise comparisons showed that Orobica had the lowest values of protein and total solids along with the highest values of lactose (*p* < 0.05); conversely, Lariana had the lowest content of lactose and the highest content of total solids (*p* < 0.05). Frisa had the highest content of both IgG and lactoferrin; Camosciata, on the contrary, showed the lowest values for these components (*p* < 0.05). As regards the fat percentage, Camosciata and Orobica had the lowest values while Lariana had the highest (*p* < 0.05).

Planned contrast “Camosciata vs. local breed” indicated that the IgG (*p* = 0.001), lactoferrin (*p* = 0.001), and fat (*p* = 0.018) concentrations of the Camosciata were lower than the average of the local breeds (Table 1).

**Figure 2:**
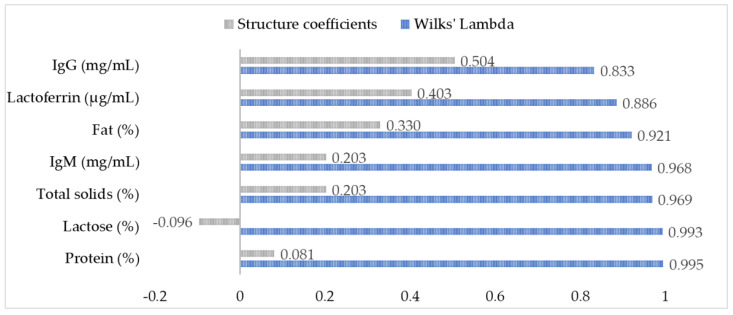
Discriminant analysis. Structure coefficients and Wilks’ lambda of colostrum component in the discriminant analysis to discriminate Camosciata from local breeds. The variables are shown in order of importance in the discrimination.

Differences between cosmopolitan and local breeds, and consequently between different farming systems, were further investigated through discriminant analysis (DA). The DA produced a model capable of discriminating Camosciata from local breeds through the characteristics of their colostrum (model Wilks’ λ = 0.559, *p* < 0.001). Figure 2 shows the variables included in the DA in order of importance according to their structure coefficients and Wilks λ. The component that contributed the most to this discrimination was IgG (*p* for λ < 0.001), followed by lactoferrin concentration (*p* for λ = 0.002) and fat percentage (*p* for λ = 0.009). The figure also shows the coefficients, which are all positive except for the lactose content. The group centroids (−1.532 for Camosciata and 0.503 for local breeds) indicated that local breeds were distinguished by a greater content of IgG, lactoferrin, fat, IgM, and total solids than Camosciata (lactose and protein had λ > 0.99 and, thus, contributed little to the discrimination).

## 4. Discussion

This study describes for the first time the colostrum composition of goat breeds reared in Northern Italy. The values we found for fat, protein, lactose, and total solids are in agreement with the literature [13,14,15,16,17,18,19,31]. However, this study also shows that significant differences in the composition of the colostrum among goat breeds exist. Specifically, regarding the chemical composition of the colostrum, Lariana showed the highest percentage of fat and total solids, while it showed the lowest percentage of lactose. On the other hand, Orobica had the lowest percentage of fat, total solids, and protein but the highest percentage of lactose. The pattern of those results was expected, considering that lactose is an osmotic active molecule. Thus, a higher percentage of it corresponds to a higher water recall and, as a consequence, a higher dilution of the other basic components of colostrum, and vice versa [51]. Our results also suggest that goat breeds which have a more pronounced meat aptitude (i.e., Frisa and Lariana) present a higher concentration of colostrum components (i.e., fat, protein, and total solids) than those with a more pronounced dairy aptitude (i.e., Orobica and Camosciata). Previous studies showed conflicting results. While Kessler et al. found few differences in colostrum composition related to the purpose for the goats, they also reported similar results to the present study for sheep [14]. In particular, these authors found that milk-type sheep breeds had lower fat and protein with concomitantly higher lactose percentages than the meat-type breeds. In goats, they only highlighted a higher IgG concentration in animals kept for meat production than in dairy breeds [14].

Similarly, in the present study, a significant difference was found in immunological parameters of the more rustic, autochthonous breeds reared with traditional systems and those of the cosmopolitan Camosciata delle Alpi. In particular, IgG is the parameter that best enables the differentiation between local and cosmopolitan breeds and thus between farming systems. The highest concentration was found in the Frisa goat; the lowest in the Camosciata. It should also be specified that in our study, total immunoglobulin G content was determined, as it was not possible to quantify the IgG subclasses (due to the lack of specific kits). The values obtained are of the same order of magnitude as those reported in the scientific literature. However, we can notice that all four breeds were in the upper limit of the range of IgG previously observed in goat colostrum [14,15,16,17,18,21,31]. In fact, Kessler et al. [14], who analyzed the colostrum of several Swiss and German goat breeds, observed an average IgG value between 4.8 and 75.0 mg/mL, with the Boer (i.e., a goat reared for meat production) showing the highest concentration. Interestingly, Levieux et al. [21] reported a highly variable range for the Camosciata (from 19.9 to 94.5 mg/mL) but this range includes the values we found for that breed.

In the present study, the IgM concentrations were also determined. They have antimicrobial properties (e.g., neutralization of viruses and agglutination of microbes) and anti-inflammatory extracellular and intracellular immune exclusion, inhibiting adherence and invasion of mucosal epithelia. Thus, IgMs can exert the first defense action in the intestinal epithelium of the newborn [52]. The scarcely available scientific literature on this molecule reports higher mean values than those found in this investigation [13,15,18]. Since our determination techniques do not differ from previous studies, the disagreement may be due to differences in breeds (i.e., Majorera and Weiße Deutsche Edelziege goats) and farming systems. However, these immunoglobulins represent a minor proportion of the total immunoglobulins in goat colostrum in relation to IgG [13]. Moreover, we did not find any differences among the evaluated breeds.

Overall, compared to previous studies, we found higher IgG and lower IgM values. However, these findings do not appear to be associated. The mechanisms exploited by these two immunological molecules are different [24], and there is no competition at the level of basal-apical transport in the alveolar cells of the mammary gland. Differences between breeds were instead found for lactoferrin concentrations. They showed the highest concentration in the Frisa goats and the lowest in Lariana and Camosciata delle Alpi. When compared to previous studies, our results exhibited much higher values, especially for goats with a more pronounced meat aptitude. Hiss et al. [30] reported a lactoferrin concentration of 387 ± 69 µg/mL in the colostrum of German Improved Fawn and White goats, while Rachman et al. [29] found a range of 156.4 to 207.4 µg/mL in Peranakan Etawah, Jawarandu, and Saanen goat crosses with Peranakan Etawah goats. In addition to breed and management differences, the varying analytical techniques used in these studies could also explain the differences in values. Regardless, in the present study, the more meat-aptitude goats also showed better results than the dairy ones in the case of the immunological quality of the colostrum. That is consistent with what was found by Kessler et al. [14] regarding several Swiss and German goat breeds, and by Altvater-Hughes et al. focusing on cattle [53].

The differences between the Camosciata delle Alpi and local breeds were pointed out by planned contrasts and discriminant analysis (DA). In particular, the DA showed that an accurate statistical model can be built for their discrimination and that IgG was the component that mostly contributed to distinguish them, followed by lactoferrin concentration and fat percentage. This discrimination may also be related to the different nutritional management of the animals enrolled in the present study. Moreover, it could be explained by two different factors linked to the evolutionary processes of the breeds. First, Camosciata has a higher milk aptitude due to the artificial selection implemented in recent decades. This high level of milk production could have led to an increase in the lactose percentage and, consequently, to a decrease in the other colostrum components. Secondly, the local breeds have been more exposed to pathogenic noxious substances than intensively reared animals during their evolutionary history. They also have a lower milk production. These factors could enhance immune components and fat concentrations in their colostrum to ensure a higher probability of survival of their offspring [20].

Some limitations should be pointed out in the current study especially related to the many factors that could influence the composition of colostrum and which have not yet been well defined. For example, the Camosciata and the local breeds differed in their rearing and nutritional systems, and it is not possible to separate the genetic aspect from the management one. Future research could develop protocols in experimental stables where farming systems and diets can be standardized and identical for all breeds. On the other hand, our study provides information on the colostrum quality which truly characterizes the goats of the Italian livestock heritage as no modifications were introduced to the typical, currently applied farming systems. Instead, we tried to limit the bias due to the parity order by enrolling only multiparous goats. Among them, the preliminary analyses indicated no difference due to their parity, but further investigations should also include primiparous goats to better understand the role of this factor in influencing colostrum quality. Regarding the possible effect of the litter size, we have found that it is associated with the breed, and thus it was not possible to include it as a covariate in the statistical models (because it would have violated an assumption of analysis). On the other hand, the number of animals did not allow an analysis within each breed. This could represent a bias, and future studies will be necessary with a larger sample size. Finally, in further research, kids could be monitored to assess the association between colostrum quality and their growth performance.

## 5. Conclusions

In conclusion, this study has shown the existence of variability in colostrum quality among local and cosmopolitan breeds of goat which could probably be linked to the different farming systems and may be explained by their human-mediated selection and natural adaptation to the environment. However, even among the autochthonous breeds we found some significant differences, which might be ascribed to different aptitudes (meat or dairy). In general, the higher quality of colostrum produced by some local goats could be associated with hardiness and rusticity as it helps the growth and survival of the kids.

## Figures and Tables

**Table 1:** Colostrum components in Camosciata and local breeds. Data are means, standard errors of the mean (SEM), and range (minimum and maximum). Lactoferrin was analyzed after logarithmic transformation, but raw data are shown. Last column reports the *p*-value for the planned contrast Camosciata vs. local breeds.

Parameter	Breed								*p* Value of ANOVA	*p* Value of the Contrast
	Camosciata		Frisa		Lariana		Orobica			
	Mean ± SEM	Range (Min–Max)	Mean ± SEM	Range (Min–Max)	Mean ± SEM	Range (Min–Max)	Mean ± SEM	Range (Min–Max)		
**Fat** **(%)**	6.92a ± 0.53	2.40–12.62	8.33ab ± 0.52	4.66–13.06	10.18b ± 0.66	3.80–16.06	7.13a ± 0.59	3.46–10.94	0.001	0.018
**Protein** **(%)**	14.16b ± 0.59	7.10–20.44	15.43b ± 0.81	8.14–23.34	16.20b ± 0.72	7.54–23.86	10.77a ± 1.07	3.52–19.78	0.001 *	0.969
**Lactose** **(%)**	2.50b ± 0.12	1.12–3.50	2.42ab ± 0.16	0.22–4.08	1.87a ± 0.17	0.14–3.18	3.16c ± 0.17	1.34–4.30	<0.001	0.959
**Total solid** **(%)**	26.37ab ± 0.62	20.90–32.84	28.76bc ± 0.99	20.10–38.36	30.73c ± 1.02	16.68–39.36	24.11a ± 1.29	15.66–35.44	0.001 *	0.100
**IgG** **(mg/mL)**	74.75a ± 4.12	34.21–106.38	100.90c ± 1.56	75.70–113.30	93.07bc ± 2.71	64.29–108.00	80.27ab ± 5.57	37.38–105.58	<0.001 *	0.001
**IgM** **(mg/mL)**	1.40 ± 0.16	0.22–2.87	1.85 ± 0.12	0.29–3.30	1.57 ± 0.13	0.41–2.75	1.44 ± 0.24	0.05–3.19	0.122 *	0.243
**Lactoferrin** **(µg/mL)**	763.10a ± 76.31	435.68–1920.03	1781.31b ± 168.69	345.35–3232.93	1148.00a ± 179.03	327.94–3321.82	1132.41ab ± 153.08	425.56–2759.11	<0.001 *	0.001

* Welch statistic. Values followed by the same letter in each row do not differ significantly (*p* < 0.05); multiple comparisons with Sidak correction.

## Data Availability

Data will be made available upon reasonable request.

## Supplementary Materials

The following supporting information can be downloaded at: https://www.mdpi.com/article/10.3390/ani13193146/s1, Table S1: Litter size per breed.

## References

[1] Scherf B., Pilling D., Scherf B.D., Pilling D. (2015). The Second Report on the State of the World’s Animal Genetic Resources for Food and Agriculture.

[2] Battaglini L., Bovolenta S., Gusmeroli F., Salvador S., Sturaro E. (2014). Environmental Sustainability of Alpine Livestock Farms. Ital. J. Anim. Sci..

[3] Bigi D., Zanon A. (2020). Atlante Delle Razze Autoctone, Bovini, Equini, Ovicaprini, Suini Allevati in Italia.

[4] Curone G., Filipe J., Cremonesi P., Trevisi E., Amadori M., Pollera C., Castiglioni B., Turin L., Tedde V., Vigo D. (2018). What We Have Lost: Mastitis Resistance in Holstein Friesians and in a Local Cattle Breed. Res. Vet. Sci..

[5] Curone G., Filipe J., Cremonesi P., Piccioli-Cappelli F., Trevisi E., Amadori M. (2019). Relevance of the Dairy Cow Biodiversity in the Development of a Profitable and Environmentally Sustainable Livestock. CAB Rev. Perspect. Agric. Vet. Sci. Nutr. Nat. Resour..

[6] ASSONAPA Associazione Nazionale Della Pastorizia. http://www.assonapa.it/.

[7] FAO Domestic Animal Diversity Information System (DAD-IS). http://www.fao.org/dad-is/en/.

[8] Agradi S., Menchetti L., Curone G., Faustini M., Vigo D., Villa L., Zanzani S.A., Postoli R., Kika T.S., Riva F. (2022). Comparison of Female Verzaschese and Camosciata Delle Alpi Goats’ Hematological Parameters in The Context of Adaptation to Local Environmental Conditions in Semi-Extensive Systems in Italy. Animals.

[9] Mann S., Curone G., Chandler T.L., Moroni P., Cha J., Bhawal R., Zhang S. (2020). Heat Treatment of Bovine Colostrum: I. Effects on Bacterial and Somatic Cell Counts, Immunoglobulin, Insulin, and IGF-I Concentrations, as Well as the Colostrum Proteome. J. Dairy Sci..

[10] Mann S., Curone G., Chandler T.L., Sipka A., Cha J., Bhawal R., Zhang S. (2020). Heat Treatment of Bovine Colostrum: II. Effects on Calf Serum Immunoglobulin, Insulin, and IGF-I Concentrations, and the Serum Proteome. J. Dairy Sci..

[11] Uruakpa F.O., Ismond M.A.H., Akobundu E.N.T. (2002). Colostrum and Its Benefits: A Review. Nutr. Res..

[12] Bergman A.J., Turner C.W. (1937). The Composition of the Colostrum of the Dairy Goat. J. Dairy Sci..

[13] Rudovsky A., Locher L., Zeyner A., Sobiraj A., Wittek T. (2008). Measurement of Immunoglobulin Concentration in Goat Colostrum. Small Rumin. Res..

[14] Kessler E.C., Bruckmaier R.M., Gross J.J. (2019). Immunoglobulin G Content and Colostrum Composition of Different Goat and Sheep Breeds in Switzerland and Germany. J. Dairy Sci..

[15] Sánchez-Macías D., Moreno-Indias I., Castro N., Morales-delaNuez A., Argüello A. (2014). From Goat Colostrum to Milk: Physical, Chemical, and Immune Evolution from Partum to 90 Days Postpartum. J. Dairy Sci..

[16] Arguello A., Castri N., Alvarez S., Capote J. (2006). Effects of the Number of Lactations and Litter Size on Chemical Composition and Physical Characteristics of Goat Colostrum. Small Rumin. Res..

[17] Romero T., Beltran M.C., Rodriguez M., Marti De Olives A., Molina M.P. (2013). Short Communication: Goat Colostrum Quality: Litter Size and Lactation Number Effects. J. Dairy Sci..

[18] Moreno-Indias I., Sánchez-Macías D., Castro N., Morales-delaNuez A., Hernández-Castellano L.E., Capote J., Argüello A. (2012). Chemical Composition and Immune Status of Dairy Goat Colostrum Fractions during the First 10h after Partum. Small Rumin. Res..

[19] Keskin M., Güler Z., Gul S., Biçer O. (2007). Changes in Gross Chemical Compositions of Ewe and Goat Colostrum during Ten Days Postpartum. J. Appl. Anim. Res..

[20] Yang M., Zou Y., Wu Z.H., Li S.L., Cao Z.J. (2015). Colostrum Quality Affects Immune System Establishment and Intestinal Development of Neonatal Calves. J. Dairy Sci..

[21] Levieux D., Morgan F., Geneix N., Masle I., Bouvier F. (2002). Caprine Immunoglobulin G, β-Lactoglobulin, α-Lactalbumin and Serum Albumin in Colostrum and Milk during the Early Post Partum Period. J. Dairy Res..

[22] Claps S., Di Napoli M.A., Caputo A.R., Rufrano D., Sepe L., Di Trana A. (2016). Factor Affecting the 3’ Sialyllactose, 6’ Sialyllactose and Disialyllactose Content in Caprine Colostrum and Milk: Breed and Parity. Small Rumin. Res..

[23] Claps S., Di Napoli M.A., Sepe L., Caputo A.R., Rufrano D., Di Trana A., Annicchiarico G., Fedele V. (2014). Sialyloligosaccharides Content in Colostrum and Milk of Two Goat Breeds. Small Rumin. Res..

[24] Zhou A., Liu G., Jiang X. (2023). Characteristic of the Components and the Metabolism Mechanism of Goat Colostrum: A Review. Anim. Biotechnol..

[25] Xu W., Mann S., Curone G. (2021). Kenéz Heat Treatment of Bovine Colostrum: Effects on Colostrum Metabolome and Serum Metabolome of Calves. Animal.

[26] Filipescu I.E., Leonardi L., Menchetti L., Guelfi G., Traina G., Casagrande-Proietti P., Piro F., Quattrone A., Barbato O., Brecchia G. (2018). Preventive Effects of Bovine Colostrum Supplementation in TNBS-Induced Colitis in Mice. PLoS ONE.

[27] Menchetti L., Traina G., Tomasello G., Casagrande-Proietti P., Leonardi L., Barbato O., Brecchia G. (2016). Potential Benefits of Colostrum in Gastrointestinal Diseases. Front. Biosci..

[28] Menchetti L., Curone G., Filipescu I.E., Barbato O., Leonardi L., Guelfi G., Traina G., Casagrande-Proietti P., Riva F., Casano A.B. (2020). The Prophylactic Use of Bovine Colostrum in a Murine Model of TNBS-Induced Colitis. Animals.

[29] Rachman A.B., Maheswari R.R.A., Bachroem M.S. (2015). Composition and Isolation of Lactoferrin from Colostrum and Milk of Various Goat Breeds. Procedia Food Sci..

[30] Hiss S., Meyer T., Sauerwein H. (2008). Lactoferrin Concentrations in Goat Milk throughout Lactation. Small Rumin. Res..

[31] Yang X.Y., Chen J.P., Zhang F.X. (2009). Research on the Chemical Composition of Saanen Goat Colostrum. Int. J. Dairy Technol..

[32] Puppel K., Gołębiewski M., Grodkowski G., Slósarz J., Kunowska-Slósarz M., Solarczyk P., Łukasiewicz M., Balcerak M., Przysucha T. (2019). Composition and Factors Affecting Quality of Bovine Colostrum: A Review. Animals.

[33] Sandrucci A., Bava L., Tamburini A., Gislon G., Zucali M. (2019). Management Practices and Milk Quality in Dairy Goat Farms in Northern Italy. Ital. J. Anim. Sci..

[34] Nicoloso L., Bomba L., Colli L., Negrini R., Milanesi M., Mazza R., Sechi T., Frattini S., Talenti A., Coizet B. (2015). Genetic Diversity of Italian Goat Breeds Assessed with a Medium-Density SNP Chip. Genet. Sel. Evol..

[35] Sponseller J.K., Steele J.A., Schmidt D.J., Kim H.B., Beamer G., Sun X., Tzipori S. (2015). Hyperimmune Bovine Colostrum as a Novel Therapy to Combat Clostridium Difficile Infection. J. Infect. Dis..

[36] Otto W., Najnigier B., Stelmasiak T., Robins-Browne R.M. (2011). Randomized Control Trials Using a Tablet Formulation of Hyperimmune Bovine Colostrum to Prevent Diarrhea Caused by Enterotoxigenic Escherichia Coli in Volunteers. Scand. J. Gastroenterol..

[37] Florén C.H., Chinenye S., Elfstrand L., Hagman C., Ihse I. (2006). ColoPlus, a New Product Based on Bovine Colostrum, Alleviates HIV-Associated Diarrhoea. Scand. J. Gastroenterol..

[38] Agradi S., Cremonesi P., Menchetti L., Balzaretti C., Severgnini M., Riva F., Castiglioni B., Draghi S., Di Giancamillo A., Castrica M. (2023). Bovine Colostrum Supplementation Modulates the Intestinal Microbial Community in Rabbits. Animals.

[39] Serra V., Castrica M., Agradi S., Curone G., Vigo D., Di Giancamillo A., Modina S.C., Riva F., Balzaretti C.M., De Bellis R. (2023). Antioxidant Activity of Different Tissues from Rabbits Fed Dietary Bovine Colostrum Supplementation. Animals.

[40] Balan P., Sik-Han K., Moughan P.J. (2019). Impact of Oral Immunoglobulins on Animal Health—A Review. Anim. Sci. J..

[41] ARAL (Associazione Regioanle Allevatori Lombardia) Razze Animali Da Reddito Allevate in Lombardia. http://old.aral.lom.it/OpuscoloRazze/index-2.html.

[42] Crepaldi P., Negrini R., Milanesi E., Gorni C., Cicogna M., Ajmone-Marsan P. (2001). Diversity in Five Goat Populations of the Lombardy Alps: Comparison of Estimates Obtained from Morphometric Traits and Molecular Markers. J. Anim.Breed. Genet..

[43] Ajmone-Marsan P., Negrini R., Crepaldi P., Milanesi E., Gorni C., Valentini A., Cicogna M. (2001). Assessing Genetic Diversity in Italian Goat Populations Using AFLP^®^ Markers. Anim. Genet..

[44] Chiatti F., Chessa S., Bolla P., Cigalino G., Caroli A., Pagnacco G. (2007). Effect of K-Casein Polymorphism on Milk Composition in the Orobica Goat. J. Dairy Sci..

[45] Associazione Formaggi Principi delle Orobie Forme. https://festivalpastoralismo.org/formaggi-orobici/principi/.

[46] Crepaldi P., Gemo G., Brambilla L., Cicogna M., Renieri C. (1999). Characterization of Val Di Livo Goat: Visible Phenotypic Profile and Body Measurements. Zootecnia e Nutrizione Animale.

[47] Higaki S., Nagano M., Katagiri S., Takahashi Y. (2013). Effects of Parity and Litter Size on the Energy Contents and Immunoglobulin G Concentrations of Awassi Ewe Colostrum. Turk. J. Vet. Anim. Sci..

[48] Field A. (2013). Discovering Statistics Using IBM SPSS Statistics.

[49] Garson G., David V. (2012). Discriminant Function Analysis.

[50] Agradi S., Curone G., Negroni D., Vigo D., Brecchia G., Bronzo V., Panseri S., Chiesa L.M., Peric T., Danes D. (2020). Determination of Fatty Acids Profile in Original Brown Cows Dairy Products and Relationship with Alpine Pasture Farming System. Animals.

[51] Fox P.F., Uniacke-Lowe T., McSweeney P.L.H., O’Mahony J.A. (2015). Dairy Chemistry and Biochemistry.

[52] Hurley W.L., Theil P.K. (2011). Perspectives on Immunoglobulins in Colostrum and Milk. Nutrients.

[53] Altvater-Hughes T.E., Hodgins D.C., Wagter-Lesperance L., Beard S.C., Cartwright S.L., Mallard B.A. (2022). Concentration and Heritability of Immunoglobulin G and Natural Antibody Immunoglobulin M in Dairy and Beef Colostrum along with Serum Total Protein in Their Calves. J. Anim. Sci..

