# Supplementary material for: Colostrum Quality in Different Goat Breeds Reared in Northern Italy
Source: Animals (Basel). 2023 Oct 9;13(19):3146. doi: 10.3390/ani13193146 (PMC10571944; doi:10.3390/ani13193146)
Supplement: Supplementary file 1 [file animals-13-03146-s001.zip › animals-2624100-supplementary.pdf]

Supplementary material

Table S1. Litter size per breed.

Number of kids	Breed				Total
	Camosciata	Frisa	Lariana	Orobica	
**1**	5_a (17.9%)	18_b (62.1%)	22_b (81.5%)	15_b (83.3%)	60 (58.8%)
**2**	21_a (75.0%)	11_b (37.9%)	5_b (18.5%)	3_b (16.7%)	40 (39.2%)
**3**	2_a (7.1%)	0_a (0.0%)	0_a (0.0%)	0_a (0.0%)	2 (2.0%)
**Total**	28 (100.0%)	29 (100.0%)	27 (100.0%)	18 (100.0%)	102 (100.0%)

Each subscript letter denotes a subset of Breed categories whose column proportions do not differ significantly from each other at the 0.05 level.
